# Pathogens of importance in lung disease—Implications of the WHO fungal priority pathogen list

**DOI:** 10.1111/resp.14623

**Published:** 2023-11-13

**Authors:** Justin Beardsley

**Affiliations:** ^1^ University of Sydney Infectious Disease Institute Sydney New South Wales Australia; ^2^ Westmead Institute for Medical Research Sydney New South Wales Australia; ^3^ Department of Infectious Diseases Westmead Hospital, NSW Health Sydney New South Wales Australia

**Keywords:** antimicrobial resistance, fungi, lung disease, public health, therapeutics

In October 2022, the World Health Organization (WHO) released its fungal priority pathogens list (FPPL).^1^ The list was the result of a global effort to systematically prioritize fungal pathogens based on their unmet research and development needs and perceived public health importance. Its aim is to focus attention and resources on these priority fungal pathogens to create new treatments, diagnostic tools and infection prevention measures. Furthermore, it aims to drive improvements in laboratory capacity, surveillance systems and public health interventions.

The WHO categorized 19 major invasive fungal pathogens into critical, high and medium priority, as shown in Table 1. Over half pose significant threats to respiratory patients. They primarily affect patients with chronic lung disease, immunocompromise or, as highlighted by the COVID‐19 pandemic, patients with critical lung infection, especially those in intensive care.^2^ It is therefore vital that respiratory physicians have a good understanding of the clinical presentations, diagnostic methods and treatment options for these infections. By promptly identifying and treating fungal infections, respiratory physicians can help to reduce morbidity and mortality and improve patient outcomes.

**TABLE 1:** WHO FPPL pathogens according to category, with pathogens of most relevance to respiratory medicine in bold.

Critical priority	High priority	Medium priority
***Aspergillus fumigatus***	***Histoplasma* spp.** ^a^	***Paracoccidioides* spp.** ^a^
***Cryptococcus neoformans***	***Fusarium* spp.**	***Coccidioides* spp.** ^a^
*Candida auris*	**Mucorales**	***Pneumocystis jirovecii***
*Candida albicans*	*Nakaseomyces glabrata* (*Candida glabrata*)	***Talaromyces marneffei*** ^a^
	Eumycetoma causative agents	***Lomentospora prolificans***
	*Candida tropicalis*	***Scedosporium* spp.**
	*Candida parapsilosis*	***Cryptococcus gattii***
		*Pichia kudriavzeveii* (*Candida krusei*)

^a^ Endemic mycoses.

The pathogens encountered will vary depending on the patient populations and the geographic region of practice, but are likely to include:

*Aspergillus fumigatus*. Primarily affects the respiratory tract of individuals with compromised immune systems or underlying lung disease. It causes diverse patterns of disease, ranging from allergic, through chronic infection (aspergilloma, chronic cavitary pulmonary aspergillosis and subacute invasive aspergillosis), to acute invasive infections.^3^, ^4^ The mainstay of therapy for this critical priority pathogen are azoles, but azole‐resistance, driven by agricultural azole use, is emerging as a major hurdle to treatment.^5^ Elevated rates of resistance of up to 20% in Europe^6^ and up to 90% in Southeast Asia^7^, ^8^ have been described and linked to poor treatment outcomes, raising red flags for the durability of current treatment guidelines.
*Cryptococcus neoformans* (critical priority) and *C. gattii* (medium priority) both cause lung infections, although infection restricted to the lung is more frequently seen with *C. gattii*. Widely viewed as opportunistic pathogens, *Cryptococcus* spp., particularly *C. gattii*, can also cause severe infections in immunocompetent hosts.^9^, ^10^ In immunocompetent patients, isolated cryptococcomas are a frequent presentation and may be asymptomatic in 25%–55% of cases.^11^, ^12^ In contrast, for people living with HIV, fulminant disease with pulmonary infiltrates may occur,^13^ and the presentation can be very hard to distinguish from other opportunistic lung infections, such as *Pneumocystis jirovecii* pneumonia (PJP) or tuberculosis, making clinical suspicion and appropriate diagnostic tests key to successful outcomes.^14^
*Histoplasma* spp. (high priority) cause life‐threatening invasive fungal infections.^15^, ^16^, ^17^ Histoplasmosis is well‐established as an endemic mycosis of the Americas^18^, ^19^ and Central and West Africa^15^, ^20^ and is increasingly recognized in Southeast Asia.^21^ Histoplasmosis affects individuals regardless of immune function or comorbidities. Although disease may be asymptomatic or mild in patients without prior immune compromise, it is often a life‐threatening progressive disseminated infection with high morbidity and mortality for those with suppressed immune function,^22^ including HIV/AIDS patients, solid organ transplant recipients or patients taking immune suppressive medications (e.g., corticosteroids or TNF‐inhibitors).^23^, ^24^, ^25^, ^26^ Mortality rates in excess of 30% have been reported among HIV patients.^19^, ^27^

The high priority moulds *Fusarium* spp. and Mucorales and the medium priority *Lomentospora prolificans* and *Scedosporium* spp. are all notable for their ability to cause local infections in immunocompetent patients and devastating systemic infections in those with immunocompromise. Although they are infrequent causes of lung infection, they present a formidable diagnostic and treatment challenges when they do occur. Many of the species are intrinsically resistant to multiple antifungal agents, resulting in complicated, toxic treatment regimens and mortality rates in excess of 50% frequently reported.

In May 2021, a COVID‐19 associated mucormycosis epidemic was declared in India, highlighting the ability of this pathogen to complicate viral respiratory infections. Over half of patients had rhino‐orbital‐cerebral infection,^28^, ^29^ and uncontrolled diabetes mellitus was the most common underlying disease.^28^ The receipt of glucocorticoid therapy for treatment of COVID‐19 was also identified as a major risk factor.^28^

The remaining medium priority fungal pathogens include those of great importance in particular patient populations or geographic ranges. *Pneumocystis jirovecii* remains a major threat to people living with HIV/AIDS (or receiving immunosuppressive therapy). Talaromycosis is a leading cause of death in HIV/AIDS in tropical and subtropical Asia, closely linked to poverty, stigma and neglect.^30^ Similarly, *Coccidioides* spp. (endemic to North, Central and South America) and *Paracoccidioides* spp. (endemic to Latin America) are important causes of pneumonia in these regions, and require greater attention.

In summary, the WHO FPPL is relevant to respiratory physicians for several reasons. First, it will help to direct their research efforts towards areas of greatest unmet need—including pathogens responsible for life‐threatening infections with limited treatment options. The development of new therapies is essential to improve patient outcomes.

Second, it highlights the need for increased clinical suspicion as well as improved diagnostic laboratory capacity. Many fungal infections are difficult to diagnose, and advocacy in both of these areas can support accurate and timely diagnoses, and ultimately improved patient outcomes for respiratory patients.

Third, the list promotes development and application of public health interventions to prevent and control fungal infections. Since many fungal infections occur in patients with chronic lung disease, it is likely to be those patients who benefit the most from such interventions. By being aware of the WHO FPPL and incorporating its recommendations into practice, respiratory physicians can play a key role in improving the overall response to fungal infections and reducing the burden of disease caused by these ubiquitous pathogens on patients and the healthcare system (Figure 1).

**FIGURE 1:**
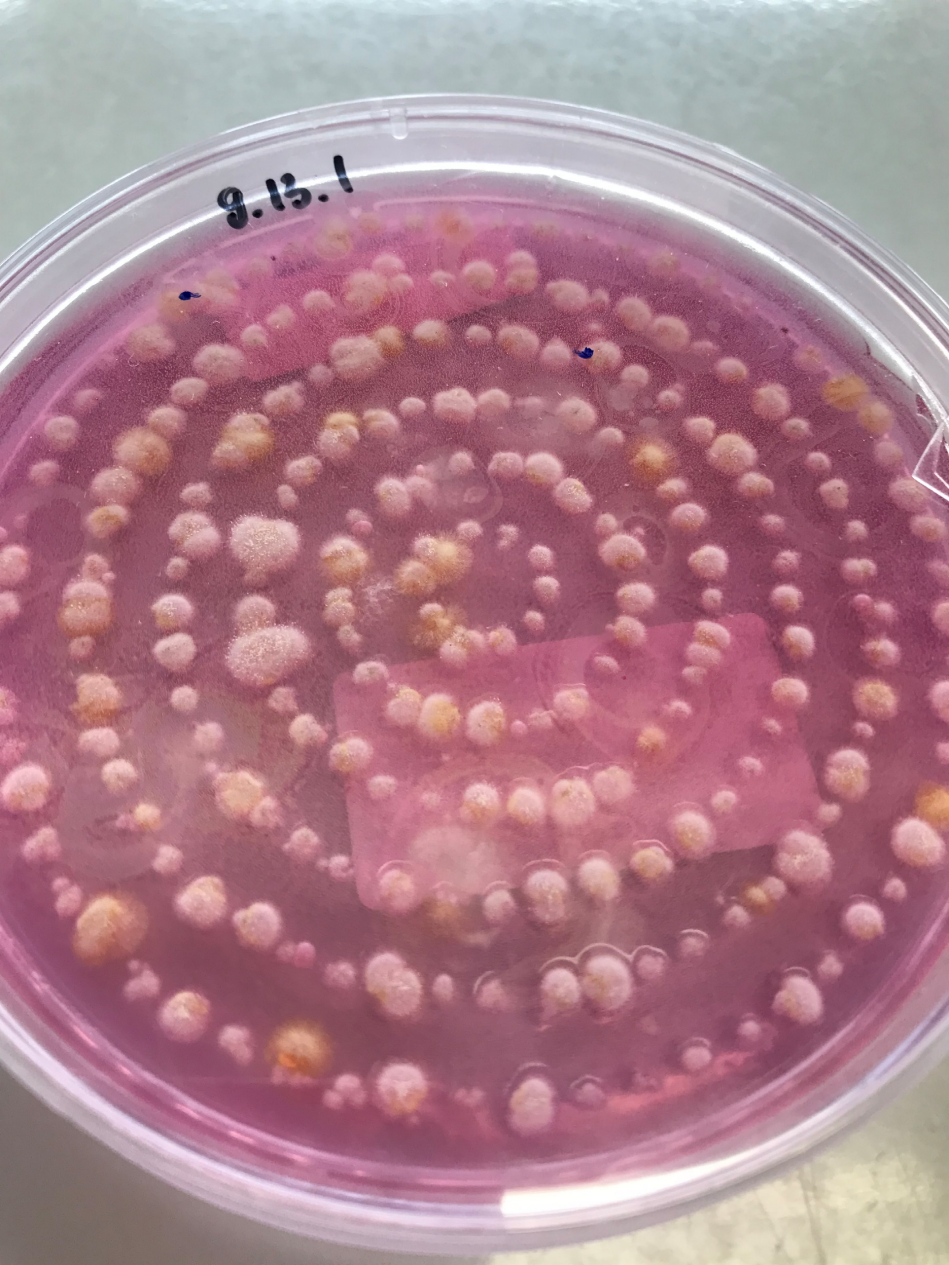
Identifying which fungi are present in the air. Assorted colonies growing on Dicholoran Rose‐Bengal Chloramphenicol agar. Image courtesy of Dr. Tra My Nu Duong.

## CONFLICT OF INTEREST STATEMENT

None declared.
